# Frailty is associated with sociodemographic and health factors and related to the care context of older caregivers: a Brazilian cross-sectional study

**DOI:** 10.1590/1516-3180.2022.0072.R1.06072022

**Published:** 2022-09-12

**Authors:** Marcela Naiara Graciani Fumagale Macedo, Élen dos Santos Alves, Isabela Thaís Machado de Jesus, Keika Inouye, Tábatta Renata Pereira de Brito, Ariene Angelini dos Santos-Orlandi

**Affiliations:** I Undergraduate Student, Department of Gerontology, Universidade Federal de São Carlos (UFSCar), São Carlos (SP), Brazil.; II MSc. Nurse and Doctoral Student, Postgraduate Nursing Program, Universidade Federal de São Carlos (UFSCar), São Carlos (SP), Brazil.; III PhD. Gerontologist and Temporary Professor, Department of Gerontology, Universidade Federal de São Carlos (UFSCar), São Carlos (SP), Brazil.; IV PhD. Pharmaceutical and Adjunct Professor, Department of Gerontology, Universidade Federal de São Carlos (UFSCar), São Carlos (SP), Brazil.; V PhD. Nurse and Adjunct Professor, Faculty of Nutrition, Universidade Federal de Alfenas (UNIFAL-MG), Alfenas (MG), Brazil.; VI PhD. Nurse and Adjunct Professor, Department of Nursing, Universidade Federal de São Carlos (UFSCar), São Carlos (SP), Brazil.

**Keywords:** Caregivers, Aged, Frailty, Geriatric nursing, Cognition, Older adults, Burden, Depressive symptoms, Frail older adult

## Abstract

**BACKGROUND:**

The task of caring can arise suddenly without guidance or support, resulting in psychological tension and health impairment, which can culminate in the development of frailty.

**OBJECTIVE:**

To analyze the relationship between frailty and sociodemographic and health aspects related to the care context of older caregivers.

**DESIGN AND SETTING:**

A cross-sectional study was conducted on 65 older caregivers registered in family health units in the interior of the state of São Paulo.

**METHODS:**

The participants were interviewed individually using the following instruments: a characterization questionnaire, Fried’s frailty phenotype, Zarit Burden’s Interview, Mini-Mental State Examination, Geriatric Depression Scale, Katz Index, and Lawton Scale. In addition, the following statistical tests were applied: Pearson’s chi-squared test, Fisher’s exact test, and Mann–Whitney test. A significance level of 5% was considered to be statistically significant.

**RESULTS:**

Women who took care of their spouses predominated without prior training or the help of other people. Most of the patients were pre-frail (72.3%). Frailty was significantly related to marital status (P = 0.016), depressive symptoms (P = 0.029), cognitive decline (P = 0.029), the degree of kinship (P = 0.015), and burden (P = 0.004).

**CONCLUSION:**

Older caregivers without a partner, with severe depressive symptoms and cognitive changes, who cared for their parents, and had higher levels of burden, presented a higher proportion of frailty.

## INTRODUCTION

In view of the formation of new family arrangements and the reduction in the number of children, older adults may be taking on the role of caregivers for other older adults. However, these older adults usually assume the task of caring suddenly, without any preparation or support, which can result in a burden. Thus, chronic stress could lead older caregivers to experience physical and emotional exhaustion, making them more vulnerable and enabling greater chances of developing frailty syndrome.^1^

Scholars point out that frailty is a relevant public health problem that deserves investigation because it has a high prevalence, its incidence increases with age, can negatively impact the quality of life of older adults and their families, and may result in undesirable adverse events that can lead to increased health care costs, in addition to high social costs.^2^ Furthermore, researchers point out that its prevalence is higher in developing countries.^3^ Thus, it is justified to conduct research related to frailty.

Studies that sought to analyze the relationship between frailty and sociodemographic and health aspects related to the care context of older caregivers are scarce in the literature and have demonstrated inconsistent results. A cross-sectional study was conducted in Campinas with 148 older caregivers recruited for health services. There was a higher chance of frailty only in the group of older caregivers with multimorbidity, regardless of the burden.^1^

In Belgium, researchers conducted a case-control study of 79 older spousal caregivers and 79 controls (older non-caregivers). The authors concluded that older spousal caregivers showed an increased risk of becoming frail, using antidepressants, and having greater difficulty maintaining their social contacts compared with that of older non-caregivers. However, the caregiver burden did not increase the chances of frailty among older caregivers.^4^ A cross-sectional study was conducted in São Carlos with 328 older caregivers from the community, which identified that 58.8% of the participants were pre-frail, and 21.1% were frail. Frailty was associated with advanced age, female sex, depressive symptoms, pain, and the absence of a partner.^5^

There are little data available in the literature on frailty syndrome in older adult caregivers and its relationship with the care context.^5^ Therefore, it is necessary to investigate it, considering that older adult caregivers with advanced age may present a higher risk of frailty^4^ because they face aging, health problems, and increasing demands related to the care process.^5^ Furthermore, in view of the above, it is relevant to know the relationship between frailty and sociodemographic and health aspects related to the care context of older caregivers, especially in poverty, since the presence of this syndrome may impair both the quality of life and well-being of these individuals.

## OBJECTIVE

To analyze the relationship between frailty and sociodemographic and health aspects related to the care context of older caregivers.

## METHODS

### Design, period, and place of study

It is an observational, cross-sectional, quantitative research, part of a larger project carried out in the city of São Carlos, state of São Paulo, Brazil, from July 2019 to March 2020, with older caregivers treated in five family health units (FHU) inserted in a context of high social vulnerability. All guidelines of the Strengthening the Reporting of Observational Studies in Epidemiology (STROBE) were included.

### Criteria of inclusion and exclusion

The study included all the older caregivers who met the following inclusion criteria: 60 years of age or older; being the primary caregiver of an older adult; being a relative of the older in care, who was dependent on at least one basic activity (BADL, evaluated using the Katz Index) or instrumental activities of daily living (IADL, assessed using the Lawton and Brody scale); informally performed the care; and living in the same household as the older being cared for, and registered at an FHU inserted in a context of high social vulnerability. The exclusion criteria were as follows: communication difficulties due to severe hearing or visual deficits, perceived at the time of data collection; being classified as independent of all older adults in the household, both for BADL and IADL; death of one of the older adults in the dyad; change of address; and not being found after three attempts of contact on different days and times.

The sample was selected from a list provided by the professionals of the five FHUs, with 168 households comprising at least two older adults. All older adults received a visit. Among them, 49 did not participate in the study, 32 were not found after three attempts of contact on different days and times, 18 no longer lived at the address reported, 3 died, and 1 of the households had independent older adults for BADL and IADL. Therefore, the final sample comprised 65 older caregivers. Considering the 168 older adult caregivers registered in the aforementioned FHUs, the 65 participants of this study constituted a sample with a degree of confidence of 95% and a margin of error of 10%, as calculated using the Survey Monkey platform.

### Study protocol

Initially, the researchers contacted the five FHUs to identify the homes to be visited. They prepared a list with the names and addresses of older caregivers with the support of nurses and community health agents in the FHUs. They then visited all older caregivers at home to verify their compliance with the inclusion and exclusion criteria. At that moment, the purpose of the study was presented, and related ethical issues and the Katz Index and Lawton Scale instruments were applied to establish who was the older caregiver and the older adult. Those who were dependent for a greater number of activities were considered older adults who received care. Researchers invited those who met the inclusion criteria to participate in the study and, in case of acceptance, scheduled a new home visit for the signing of the informed consent statement and the beginning of the evaluation. Eight previously trained undergraduate and graduate students performed data collection, which took place in the residence of the older adults individually and lasted approximately 2 hours.

The researchers prepared a questionnaire and applied it to characterize older caregivers and their care context. The information included sex, age, marital status, education, race/color, retirement, family and individual income, number of people residing in the home, private health plan, number of medications in use, multimorbidity, pain, degree of kinship, how long the care was exercised, how many hours and days in the week they spent on this care, if they underwent any preparatory course, and if they received help for the task of caring. In addition, the following instruments were used.

To evaluate frailty in older caregivers, the study adopted the phenotype proposed by Linda Fried based on five elements:

1. Unintentional weight loss – “Do you think you have lost weight without dieting in the last 12 months?” If yes, if this weight loss was equal to or greater than 4.5 kg or 5% of body weight in the previous year, the older adults scored this criterion.
2. Fatigue – “How often did you feel that everything you did required a lot of effort in the last week”? and “How often did you feel that you could not carry on with your things in the last week”? The older adults who answered “always” or “most of the time” for either of these two questions scored on this criterion.
3. Low palmar grip strength: The researchers measured it using a portable hydraulic dynamometer in the dominant hand. They performed three consecutive measurements of palmar grip strength using the arithmetic mean. Then, the results were adjusted to complete the criteria according to sex and body mass index (BMI).
4. Low level of caloric expenditure: adapted question. It was assessed by self-report based on the following question: “Do you think you perform less physical activity than you did 12 months ago?” If so, older adults scored this criterion.
5. Slow gait: This is indicated by the average time spent traveling a distance of 4.6 m, with adjustments according to sex and height. Three gait speed measurements were performed using the arithmetic mean. The presence of three or more of the five characteristics of the phenotype indicates a frail older adult; one or two means pre-frailty, and none of these characteristics indicates robust or non-frail older adults.^6^

The study used the Katz Index and Lawton and Brody scale to evaluate the functional capacity of both older caregivers and adults who receive care. The Katz Index for BADLs was validated for the Brazilian context and was used to evaluate six areas of everyday life of self-care and presented as response options: “independent” or “dependent.” Ultimately, the interviewer should check the number of activities the older adults are independent in and how many activities they are dependent on.^7 ^Lawton and Brody’s scale of IADLs aimed to evaluate the degree of independence of the interviewee in seven activities. The final score can vary between 7 and 21 points; 7 points indicate total dependence, 8 to 20 points indicate partial dependence, and 21 points indicate independence.^8^

The research used the Zarit Burden Interview to assess the burden of older caregivers, consisting of 22 Likert questions. The result obtained at the end of the questionnaire was the sum of all the questions answered, ranging from 0 to 88 points. The higher the score obtained, the greater the burden perceived by the caregiver.^9 ^The cut-off point suggested using the international study by Ferreira et al.:^10 ^little burden (0–20), moderate burden (21–40), moderate to severe burden (41–60), and severe burden (61–88).

The Mini-Mental State Examination was used to assess cognition in older caregivers. The instrument consisted of 12 items with a maximum score of 30 points. The cut-off score adopted to indicate possible evidence of cognitive impairment varies according to the respondent’s education, of which 17 points were for illiterate, 22 points for individuals with 1–3 years of education, 24 points for 4–7 years of study, and 26 points for people with 8 years of education or more.^11^

Researchers used the Geriatric Depression Scale to screen depressive symptoms in older caregivers, a 15-items version. Finally, we used it to calculate the sum of the scores obtained. A score between 0 and 5 points indicates an absence of depressive symptoms, 6 to 10 points indicate mild depressive symptoms, and 11 to 15 points indicate severe depressive symptoms.^12^

### Ethical aspects

The study followed all the ethical aspects contained in Resolution 466/12. Data collection began only after authorization from the Municipal Health Department and subsequent approval from the ethics committee of Human Research of the Universidade Federal de São Carlos (UFSCar) [CAAE:08175419.5.0000.5504; number 3.275.704; approval date: April 22, 2019].

### Analysis of results and statistics

The Kolmogorov–Smirnov test was used to verify the normality of the variables. It helped to estimate the frequency distributions, means, and minimum and maximum values for the numerical variables of the study for descriptive analysis of the data. The proportions of categorical variables were also estimated. Pearson’s chi-square, Fisher’s exact, and Mann–Whitney tests were used to identify differences between the groups. Pearson’s chi-square test or Fisher’s exact test was used to compare categorical variables, which were sociodemographic and health characteristics of the frailty groups. The Mann–Whitney test was used to compare numerical variables: care time, daily hours, and weekly day of care in the frailty groups. A significance level of 5% was used. The data obtained were encoded and typed in an electronic spreadsheet using two different digitizers and analyzed with the support of the statistical package Stata (version 13.0; StataCorp, College Station, United States).

## RESULTS

The study sample consisted of 65 older caregivers. Of these, 72.3% were pre-frail, 24.6% were frail, and 3.1% were robust. Regarding frailty criteria, reduction in physical activity was the most prevalent component (75.4%), followed by fatigue (38.5%), weakness (35.4%), weight loss (24.6%), and slow gait (21.5%). Table 1 presents the sociodemographic characteristics of the older caregivers in the context of high social vulnerability according to frailty.

**Table 1.** Sociodemographic characteristics of older caregivers in a context of high social vulnerability according to frailty in the city of São Carlos, state of São Paulo (SP), Brazil, 2019–2020 (n = 65)

Variables	n(%)	Frailty		P
		No n(%)	Yes n(%)	
**Sex**						
Male	28 (43.1)	20 (71.4)	8 (28.6)	0.520
Female	37 (56.9)	29 (78.4)	8 (21.6)	
**Age group**						
60 to 74 years	51(78.5)	39 (76.5)	12 (23.5)	0.698
75 years or older	14 (21.5)	10 (71.4)	4 (28.6)	
**Marital status**						
With companion	61 (93.9)	48 (78.7)	13 (21.3)	0.016
No companion	4 (6.1)	1 (25.0)	3 (75.0)	
**Retired**						
No	17 (26.2)	14 (82.4)	3 (17.6)	0.438
Yes	48 (73.8)	35 (72.9)	13 (27.1)	
**Years of study**						
Medium (min–max)	3 (0-14)	3 (0–11)	4 (0–14)	0.950
**Race/color**						
White	21 (32.3)	19 (90.5)	2 (9.5)	0.098
Black	7 (10.8)	5 (71.4)	2 (28.6)	
Brown	34 (52.3)	24 (70.6)	10 (29.4)	
Indigenous people	1 (1.5)	0 (0.0)	1 (100.0)	
Yellow	2 (3.1)	1 (50.0)	1 (50.0)	
**Personal income**						
Medium (min–max)	998 (0–6.000)	998 (0–6.000)	998 (0–2.700)	0.379
**Family income**						
Medium (min–max)	2.090 (300–6.998)	2.000 (300–6.998)	2.094 (980–3.700)	0.949
**Number of residents in the household**						
Medium (min–max)	2 (2–9)	2 (2–5)	2 (2–9)	0.372

The proportion of frailty was higher among older caregivers who did not have a partner than among those with a partner (P = 0.016). Table 2 presents the health characteristics of older caregivers in the context of high social vulnerability according to frailty.

**Table 2.** Health characteristics of older caregivers in a context of high social vulnerability according to frailty in the city of São Carlos, state of SP, Brazil, 2019–2020 (n = 65)

Variables	n (%)	Frailty		P
		No n (%)	Yes n (%)	
**Health care plan**				
No	56 (86.2)	44 (78.6)	12 (21.4)	0.207
Yes	9 (13.8)	5 (55.6)	4 (44.4)	
**Pain**				
No	6 (9.2)	4 (66.67)	2 (33.33)	0.631
Yes	59 (90.8)	45 (76.27)	14 (23.73)	
**Multimorbidity**				
No	3 (4.6)	3 (100.0)	0 (0.0)	0.311
Yes	62 (95.4)	46 (74.2)	16 (25.8)	
**Use of medicines**				
None	8 (12.3)	6 (75.0)	2 (25.0)	0.107
A	10 (15.4)	10 (100.0)	0 (0.0)	
Two or more	47 (72.3)	33 (70.2)	14 (29.8)	
**Depressive symptoms**				
Severe	5 (7.7)	2 (40.0)	3 (60.0)	**0.029**
Light	15 (23.1)	9 (60.0)	6 (40.0)	
Absence	45 (69.2)	38 (84.4)	7 (15.6)	
**Cognitive decline**				
No	18 (27.7)	17 (94.4)	1 (5.6)	**0.029**
Yes	47 (72.3)	32 (68.1)	15 (31.9)	
**BADL**				
Independence	48 (73.9)	36 (75.0)	12 (25.0)	1.000
Dependence on an activity	16 (24.6)	12 (75.0)	4 (25.0)	
Dependence on two activities	1 (1.5)	1 (100.0)	0 (0.00)	
**IADL**				
Partial dependence	41 (63.1)	29 (70.7)	12 (29.3)	0.373
Independence	24 (36.9)	20 (83.3)	4 (16.7)	

BADL = Basic Activities of Daily Living; IADL = Instrumental Activities of Daily Living.

The study indicated that the proportion of frailty was higher among older caregivers with severe depressive symptoms when compared with that of the others (P = 0.029). Statistically significant differences were also identified between cognitive decline and frailty (P = 0.029). Among older caregivers with cognitive changes, the percentage of frailty was higher when compared with that of older adults without cognitive impairment. Table 3 presents the characteristics related to the care conditions of older caregivers in the context of high social vulnerability according to frailty.

**Table 3.** Characteristics related to the care context of older caregivers in a context of high social vulnerability according to frailty in the city of São Carlos, state of SP, Brazil, 2019–2020 (n = 65)

Variables	n (%)	Frailty		P
		No n (%)	Yes n (%)	
**Who gets the care**				
Spouse	58 (89.3)	46 (79.3)	12 (20.7)	**0.015**
Parent	3 (4.6)	0 (0.0)	3 (100.0)	
Father-in-law/mother-in-law	1 (1.5)	1 (100.0)	0 (0.0)	
Brother/sister	1 (1.5)	1 (100.0)	0 (0.0)	
Others	2 (3.1)	1(50.0)	1 (50.0)	
**Care time (years)**				
Medium (min–max)	5.0 (0.05–50)	5.0 (0.05–50)	8.0 (0.5–45)	0.799
**Daily hours of care**				
Medium (min–max)	24 (1–24)	24 (1–24)	24 (2–24)	0.549
**Weekly days of care**				
Medium (min–max)	7 (4-7)	7 (4–7)	7 (7–7)	0.415
**Previous training**				
No	63 (96.9)	47 (74.6)	16 (25.4)	1.000
Yes	2 (3.1)	2 (100.0)	0 (0.0)	
**Get help in care**				
No	38 (58.5)	29 (76.3)	9 (23.7)	0.836
Yes	27 (41.5)	20 (74.1)	7 (25.9)	
**Burden**				
Small	27 (41.5)	24 (88.9)	3 (11.1)	**0.004**
Moderate	27 (41.5)	21 (77.8)	6 (22.2)	
Moderate to severe	9 (13.9)	4 (44.4)	5 (55.6)	
Severe	2 (3.1)	0 (0.00)	2 (100.0)	

min–max = minimum–maximum.

The results showed that the proportion of frailty was higher among older adults who cared for their parents when compared with that of other categories (P = 0.015). Moreover, the majority of the older caregivers who scored for the absent or moderate burden were not frail, while older caregivers who scored for moderate to severe or severe burns were frail (P = 0.004).

## DISCUSSION

Older pre-frail caregivers were predominant (72.3%), followed by frail caregivers (24.6%). Although they present different proportions, it also identified a higher prevalence of pre-frailty in a national survey conducted with caregivers of older adults from São Paulo municipalities.^1^ This divergence in proportion may have occurred because of the use of different instruments to assess frailty. An international study also observed a predominance of older pre-frail caregivers.^4^

Being an older caregiver may favor their entry into the cycle of frailty because of the greater exposure to stressors due to aging associated with the presence of morbidities. In addition, the older caregiver undergoes intense changes in their daily routine that can negatively reflect their physical and psychological health, making them more vulnerable to adversity, which would facilitate the installation of the syndrome.^5^ Researchers point out that the risk of an older caregiver becoming frail may be partially related to the lower propensity of these caregivers to engage in preventive health behaviors.^4^

The present study found that older caregivers who did not have a partner had a higher proportion of frailty. A case-control study conducted in Belgium showed divergent data.^4^ However, a national investigation conducted with 328 older caregivers identified that participants without partners had 11.03 and 14.39 times more chances of developing pre-frailty and frailty, respectively, when compared with those with partners.^5^

According to the literature, being married is a positive condition for general health. Evidence shows that having a partner raises the feeling of well-being, works as a protective factor against loneliness, and exposes the couple to healthier lifestyle habits, which are extremely important for physical, mental, and cognitive maintenance. Therefore, older adults without a partner can become frail due to insufficient physical activity and food inadequacy, which are factors that contribute to sarcopenia.^13^

In the present study, the proportion of frailty was higher among older caregivers with severe depressive symptoms. The data we found corroborate those of other investigations.^5,13,15^ Scholars state that older adult caregivers may manifest more depressive symptoms than non-caregivers and that a possible explanation would be the high demand for care and emotional pressure derived from the solitary performance of the task of caring.^16^ Evidence from the literature indicates similarity in the pathophysiological changes of both conditions, which suggests a bidirectional relationship between frailty and depression.^17^

Older adults with severe depressive symptoms demonstrate a lack of energy, reduced physical activity, and inappetence, which are recognized as open doors to the cycle of frailty. On the other hand, frail older adults may also present depressive symptoms in the presence of multimorbidity and possible functional limitations that may arise.^18^

Older caregivers with cognitive impairment had a higher proportion of frailty than those without cognitive impairment. Scholars have dedicated themselves to investigating a new condition conceptualized as cognitive frailty, identified based on cognitive impairment related to the criteria of frailty syndrome without the diagnosis of neurodegenerative diseases.^19^

There is also controversy among researchers regarding the inclusion of cognitive impairment as one of the criteria for frailty,^19^ given that both conditions are multifactorial, have a higher incidence in older adults, and seem to share similar pathophysiological mechanisms. In addition, female sex, low education, and sedentary lifestyle are possible risk predictors for both conditions.^20^

From this perspective, the context of high social vulnerability can be configured as a risk factor for older caregivers because the profile of these individuals reveals low education, financial and support scarcity, greater exposure to psychosocial stressors, and low adherence to the treatment of diseases, as well as physical and cognitive impairments.^21^ Thus, cognitive impairment due to physical conditions has the potential for reversibility. Therefore, the sooner interventions are identified, the more effective they may be.^22^

Older adults who cared for their parents presented a higher percentage of frailty than that of the others. Scholars point out that the responsibility of care can be considered an obligation, given that parents have previously devoted care to their children.^23^ Thus, the feeling of obligation combined with uninterrupted care could trigger symptoms of exhaustion and lack of time for oneself, discouraging older adults from performing leisure activities and increasing the possibility of falling within the criteria of the phenotype.^5^ This explanation is in line with the context of care in the present study.

In the present study, most older caregivers who scored little or moderate burden were not frail, while most of those who scored moderate to severe or severe burden were frail. These findings confirm the results of a national study.^1,14^ Assuming the role of caregiver of another older adult requires a high degree of vigilance and attention, which can generate physical and psychological tension over time, especially in the face of unpredictable situations and the lack of social support.^1^ In addition, cohabiting with the recipient of care exposes the caregiver to continuous and uninterrupted demand, favoring low insertion in social, physical, and leisure activities.^21^ Such conditions interact with each other, leading to the entry of older adults into the cycle of frailty. 

Frailty syndrome results from a series of changes in biological mechanisms that culminate in the deregulation of multiple systems and, consequently, in homeostatic imbalance. Therefore, the body cannot tolerate stressors in the face of the reduced available energy, which triggers a progressive decline in physical functioning,^24^ contributing to the individual feeling overwhelmed. In contrast, burns can also cause homeostatic imbalance and favor the occurrence of frailty, considering that, in the care context, there are high demands and an excess of tasks that need to be performed. It could generate a feeling of fatigue and exhaustion in addition to the short time for self-care, which would contribute to physical inactivity, a known path to the cycle of frailty. Thus, this explanation route has a double meaning; that is, a weakened organism may have a more impactful view of the care context, leading to a higher perception of burden, just as a burdened individual may present dysfunction of multiple systems and become frail.

In the face of such reflections, this study suggests that primary healthcare professionals should develop psychosocial and psychoeducational actions that aim to reduce the impact of tension involved in the task of care. Therefore, group interventions can contribute to the exchange of experiences, stimulate social interaction, and offer support to caregivers inserted in this context^25^ since the absence of support and education can subject the older caregiver to the worsening of already installed morbid conditions. Integrating health promotion and disease prevention behaviors can prevent the burden from being added to other occurrences, thus reducing the chances of the caregiver becoming frail and presenting unfavorable health outcomes, such as falls, early institutionalization, hospitalization, and death.^1^

The study recommends the development of new research with this theme since high levels of burden can culminate in the development of the syndrome and lead to adverse outcomes. Thus, intervention studies can contribute to minimizing the impact of burden and frailty in older caregivers. The inclusion of new variables, such as social support, can enhance a more comprehensive understanding of the profile of older adults and fill gaps in the literature.

The present study has some limitations. The cross-sectional design did not allow us to assign causality between variables. In addition, the small sample size and the specific context of the social vulnerability of older caregivers limit the generalization of the findings.

## CONCLUSION

Older caregivers without a partner, with severe depressive symptoms and cognitive changes, who cared for their parents, and had higher levels of burden, presented a higher proportion of frailty.
